# Long-Term Exposure to Particulate Matter 2.5 and Ozone and the Risk of Acute Respiratory Infections: Community-Based Prospective Cohort Study

**DOI:** 10.2196/88045

**Published:** 2026-07-13

**Authors:** Xiaolei Duan, Xiao Yu, Hongyu Liang, Zexuan Wen, Wenyong Zhou, Ying Ma, Weibing Wang, Yaxu Zheng, Huanyu Wu

**Affiliations:** 1 Department of Epidemiology, School of Public Health, Fudan University, Shanghai, China; 2 Institute for Surveillance and Early Warning, Shanghai Municipal Center For Disease Control Prevention, Shanghai, China; 3 Shanghai Institute of Infectious Disease and Biosecurity, Fudan University, Shanghai, China; 4 Key Laboratory of Public Health Safety of Ministry of Education, Fudan University, Shanghai, China; 5 Key Laboratory of Health Technology Assessment, Fudan University, Shanghai, China; 6 IRDR-ICoE on Risk Interconnectivity and Governance on Weather/Climate Extremes Impact and Public Health, Fudan University, Shanghai, China; 7 Shanghai Municipal Center for Disease Control and Prevention, No. 1399 Shenhong Road, Xinhong Subdistrict, Shanghai, 201107, China, +86-62758710

**Keywords:** acute respiratory infections, particulate matter, ozone, influenza-like illness, cohort study

## Abstract

**Background:**

Acute respiratory infections (ARIs) remain a major global health concern. Although long-term air pollution exposure has been linked to ARIs, prospective evidence from community-based populations remains limited.

**Objective:**

This study aimed to quantify the burden of ARIs in the community and examine the associations between long-term exposure to particulate matter 2.5 (PM_2.5_) and ozone (O_3_) and the risks of ARIs, with additional analyses using influenza-like illness (ILI) as a more specific outcome.

**Methods:**

We conducted a prospective cohort study including 3617 residents in Shanghai, China, who were followed weekly for 1 year. Individual-level exposure to PM_2.5_ and O_3_ concentrations was estimated using high-resolution datasets. Cox proportional hazards models with shared frailty were applied to assess associations with ARIs. Exposure windows of 3, 6, 9, and 12 months were evaluated, and the optimal window was selected based on the Akaike information criterion. Effect estimates were reported per IQR increase. Dose-response relationships, subgroup analyses, and multiple sensitivity analyses were performed.

**Results:**

During 3217 person-years of follow-up, 885 ARI events were documented (0.27 per person-year). In the fully adjusted model using the 12-month exposure window, each IQR increase in PM_2.5_ was associated with higher risks of ARIs (hazard ratio [HR] 1.594, 95% CI 1.340‐1.897), with stronger associations observed for ILI (HR 1.948, 95% CI 1.484‐2.557). For O_3_, the corresponding HRs were 1.510 (95% CI 1.135‐2.007) for ARIs, with stronger associations for ILI (HR 2.229, 95% CI 1.385‐3.588). PM_2.5_ showed a nonlinear association with ARIs, whereas linear relationships were observed for PM_2.5_ with ILI and O_3_ with both outcomes. Evidence of effect modification was observed by age, residence, and season for PM_2.5_ and by season for O_3_. Results were robust across multiple sensitivity analyses.

**Conclusions:**

Long-term exposure to PM_2.5_ and O_3_ is associated with increased risk of ARIs, with similar associations observed for ILI. These findings highlight the importance of long-term air pollution control and targeted interventions for susceptible populations, particularly during cold seasons.

## Introduction

Acute respiratory infections (ARIs) are among the most common infectious diseases worldwide and remain a serious public health issue [1]. In 2021, an estimated 12.8 billion upper respiratory infections and 344 million lower respiratory infections (LRIs), excluding COVID-19, occurred globally [2,3]. The etiology of ARIs is multifactorial, involving a broad spectrum of pathogens, including viruses, bacteria, and atypical organisms [4]. In addition, host factors such as age, immune status, socioeconomic conditions, and environmental determinants influence the susceptibility to and severity of ARIs [5-7]. Among environmental exposures, air pollution—particularly particulate matter (PM) and ozone (O_3_)—has been widely recognized as an important external factor influencing the risk of ARIs [8,9]. According to the World Health Organization (WHO), nearly 6.67 million premature deaths worldwide each year are attributable to air pollution, including approximately 4.14 million and 0.37 million deaths attributed to PM and O_3_, respectively [10].

Previous studies have shown that long-term exposure to air pollution is associated with an increased risk of ARIs [11-13]. In Spain, each IQR increase in PM_2.5_ and O_3_ was associated with 10% and 3% higher risks of LRIs, respectively [11]. However, most existing studies have relied on outpatient or inpatient data, which may underestimate the true burden of ARIs in the general population, as many mild cases do not seek medical care [14]. In addition, most studies have focused on first-onset events, while evidence on recurrent ARIs remains limited. Only a few studies have examined recurrent respiratory infections, often using clinical records, which may still miss a substantial proportion of community-level cases [15]. Furthermore, frailty models account for within-individual correlation and unobserved heterogeneity, providing more reliable risk estimates for recurrent events [16].

This study aimed to quantify the community burden of ARIs and to evaluate the associations between long-term exposure to PM_2.5_ and O_3_ and the risk of ARIs in a community-based cohort. By integrating high-frequency follow-up data with individual-level exposure assessment, this study provides insights into the community burden of ARIs and the potential health effects of long-term air pollution exposure. These findings may inform targeted interventions and public health strategies to reduce the burden of ARIs in community settings.

## Methods

### Study Population

A stratified random sampling design was used to ensure representative coverage of Shanghai’s central urban and suburban regions. Two central urban districts (Jing’an and Xuhui) and 3 suburban districts (Jiading, Songjiang, and Fengxian) were selected based on geographic distribution and socioeconomic characteristics. Within each selected district, subdistricts (urban) or towns (suburban) were randomly sampled. Households were then recruited through local community health centers using a probability proportional to size method based on data from the 2020 Shanghai Census, and all household members were enrolled in the cohort. The detailed design has been described previously [17].

Participants were eligible if they had resided in Shanghai for ≥6 months and provided written informed consent. Individuals with ARIs within 2 weeks before enrollment or with a diagnosis of immunodeficiency or malignancy were excluded. The final sample was demographically representative of the general population, thereby minimizing potential selection bias (Table S1 in Multimedia Appendix 1).

### Data Collection

In May 2024, a questionnaire survey was administered at community health service centers to collect individual-level information on sociodemographic characteristics and lifestyle factors, including age, sex, height, weight, smoking status, alcohol consumption, mask-wearing frequency, and vaccination history (Table S2 in Multimedia Appendix 1). Residential environmental data were also collected, including household address, fuel type, and exposure to cooking fumes.

In addition, city-level influenza-like illness (ILI) activity was obtained from sentinel hospital surveillance systems, defined as the proportion of ILI cases among all outpatient and emergency visits. For each participant, season was assigned based on the time of event occurrence (or the last follow-up date for noncases), with May to October defined as the warm season and November to April as the cold season.

### Ethical Considerations

This study was approved by the Ethics Committee of the Shanghai Center for Disease Control and Prevention (KY-2024‐19). Written informed consent was obtained from all participants before enrollment.

### Environmental Exposure

Daily PM_2.5_ concentrations and O_3_ levels, defined as the maximum daily 8-hour average, were obtained from the Tracking Air Pollution in China dataset, with spatial resolutions of 1 km×1 km and 10 km×10 km, respectively [18]. Daily temperature and relative humidity data were obtained from the ERA5-Land reanalysis dataset provided by the European Centre for Medium-Range Weather Forecasts, with a spatial resolution of 0.1°×0.1° (approximately 10 km×10 km; [19]). Individual exposure levels were estimated using bilinear interpolation based on the 4 nearest grid points surrounding each participant’s geocoded residential address [20].

Air pollution data were additionally obtained from the China National Environmental Monitoring Center (19 monitoring stations in Shanghai), and meteorological data were obtained from the Global Historical Climatology Network Daily dataset provided by the National Centers for Environmental Information (3 meteorological stations) for sensitivity analyses (Figure S1 in Multimedia Appendix 1).

### Outcome Definition

ARIs were defined according to previous studies as the acute onset of at least 1 respiratory symptom (eg, cough, sputum, sore throat, nasal congestion, rhinorrhea, anosmia, ageusia, shortness of breath, chest pain, tachypnea, or respiratory distress) together with at least 1 systemic symptom (fever, chills, hypothermia, or myalgia) [17,21]. Prior to data collection, general practitioners and community health workers received standardized training. Weekly telephone follow-ups were conducted every Thursday over a 52-week period (through May 2025). For each self-reported episode, trained staff recorded symptom type, onset date, recovery date, and health care use to ascertain ARIs.

For participants with recurrent episodes but missing recovery dates, recovery was assumed to occur 14 days after symptom onset, as ARIs are typically self-limited illnesses, with most symptoms resolving within 1 to 2 weeks [22]. Alternative recovery durations of 7 and 21 days were further evaluated in sensitivity analyses. Considering the pronounced seasonal pattern of ARIs, all participants were followed for 1 year. Participants who were unreachable for 2 consecutive follow-ups were classified as lost to follow-up. In addition, a stricter definition of ILI was applied, defined as ARIs with a body temperature ≥38.0 °C [23].

### Statistical Analyses

#### Descriptive Analyses

Continuous variables with nonnormal distributions were summarized as medians with IQRs, and group differences were compared using the Wilcoxon rank-sum test. Categorical variables were presented as frequencies and percentages, and differences between groups were assessed using Pearson chi-square test. Missing covariate data were handled using multiple imputation by chained equations.

#### Exposure Modeling

Given the strong within-individual correlation of recurrent ARIs, Cox proportional hazards models with frailty terms were applied to account for unobserved individual heterogeneity, an approach considered appropriate for recurrent event data [24]. Participants who experienced an event remained at risk for subsequent events until censoring, allowing for recurrent event analysis. Schoenfeld residual tests were used to assess the proportional hazards assumption (Table S3 in Multimedia Appendix 1). Covariates were selected to minimize confounding while avoiding overadjustment, based on a priori knowledge and univariable analyses (Table S4 in Multimedia Appendix 1). The adjusted model included age, sex, residence, household size, BMI category, smoking status, alcohol consumption, mask-wearing frequency, underlying disease, vaccination history, household fuel type, cooking fume exposure, season, and city-level ILI activity.

As the optimal exposure window for long-term air pollution remains uncertain, we evaluated multiple exposure windows of 3, 6, 9, and 12 months based on previous studies [11,13]. Given that this study involved recurrent event data, C-index and receiver operating characteristic–based approaches were not applicable. Therefore, model comparison was primarily conducted using Akaike information criterion. The primary analysis was conducted using the optimal exposure window, and effect estimates were reported per IQR increase in exposure. Four sequential models were further constructed: a single-pollutant model, a copollutant model (mutually adjusting PM_2.5_ and O_3_), a model additionally adjusted for temperature, and a model further adjusted for relative humidity. Variance inflation factors were calculated to assess collinearity among pollutants and meteorological variables (Table S5 in Multimedia Appendix 1).

#### Dose-Response and Subgroup Analyses

Restricted cubic spline models were used to assess the dose-response relationships between long-term air pollution exposure and ARIs, as well as ILI, with reference values based on the WHO guidelines (PM_2.5_: 15 μg/m^3^; O_3_: 100 μg/m^3^). Subgroup analyses were conducted stratified by age (<18, 18‐59, and ≥60 years), sex (male and female), residential area (central urban and suburban), smoking status (never and ever), and season (cold season and warm season) to explore potential effect modification.

#### Sensitivity Analyses

Several sensitivity analyses were conducted to assess the robustness of the results: (1) alternative assumptions for recovery time (7-day and 21-day definitions); (2) Andersen-Gill models for recurrent events, which provide robustness under different assumptions regarding within-individual correlation (Table S6 in Multimedia Appendix 1); (3) use of ground monitoring station data instead of satellite-based exposure estimates; and (4) exposure estimates weighted by individual time-activity patterns in a subcohort (n=1487), allowing for refined personal exposure assessment.

All analyses were performed using R software (version 4.5.0; R Foundation for Statistical Computing). A 2-sided *P* value <.05 was considered statistically significant.

## Results

### Descriptive Characteristics

A total of 3617 participants were included, with a median age of 44.1 (IQR 31.6‐57.7) years and a balanced sex distribution. Most participants had normal BMI (n=2264, 62.6%), had never smoked (n=2881, 79.7%) or consumed alcohol (n=2790, 77.1%), and used clean household fuel (n=3295, 91.1%; Table 1). During the follow-up period spanning 3217 person-years, a total of 885 ARI events were documented, corresponding to an overall incidence rate of 0.27 per person-year. Compared with participants without ARIs, those who experienced ARIs tended to be younger, female, and reside in suburban areas. Participants with ILI showed similar demographic patterns.

**Table 1.** Baseline characteristics and acute respiratory infection (ARI) incidence among participants.

Characteristic	Overall (N=3617)	Non-ARI (n=3049)	ARI (n=568)	*P* value^a^	ILI^b^ (n=189)
Age (years), median (IQR)	44.1 (31.6‐57.7)	45.7 (33.0‐59.3)	36.4 (25.7‐48.2)	<.001	34.2 (16.9‐48.1)
Age (years), n (%)				<.001	
<18	447 (12.4)	332 (10.9)	115 (20.2)		48 (25.4)
18‐59	2377 (65.7)	1980 (64.9)	397 (69.9)		123 (65.1)
≥60	793 (21.9)	737 (24.2)	56 (9.9)		18 (9.5)
Sex, n (%)				<.001	
Male	1803 (49.8)	1573 (51.6)	230 (40.5)		74 (39.2)
Female	1814 (50.2)	1476 (48.4)	338 (59.5)		115 (60.8)
Residence, n (%)				.004	
Central urban	1075 (29.7)	935 (30.7)	140 (24.6)		42 (22.2)
Suburban	2542 (70.3)	2114 (69.3)	428 (75.4)		147 (77.8)
Household size, n (%)				.30	
1	330 (9.1)	275 (9.0)	55 (9.7)		12 (6.3)
2	954 (26.4)	818 (26.8)	136 (23.9)		44 (23.3)
≥3	2333 (64.5)	1956 (64.2)	377 (66.4)		133 (70.4)
BMI category (kg/m^2^), n (%)				.06	
Underweight	293 (8.1)	232 (7.6)	61 (10.7)		26 (13.8)
Normal	2264 (62.6)	1923 (63.1)	341 (60.0)		112 (59.3)
Overweight	829 (22.9)	703 (23.1)	126 (22.2)		37 (19.6)
Obese	231 (6.4)	191 (6.3)	40 (7.0)		14 (7.4)
Smoking status, n (%)				<.001	
Never	2881 (79.7)	2386 (78.3)	495 (87.1)		173 (91.5)
Ever	736 (20.3)	663 (21.7)	73 (12.9)		16 (8.5)
Alcohol consumption, n (%)				.003	
Never	2790 (77.1)	2325 (76.3)	465 (81.9)		161 (85.2)
Ever	827 (22.9)	724 (23.7)	103 (18.1)		28 (14.8)
Mask-wearing frequency, n (%)				.006	
Never	508 (14.0)	429 (14.1)	79 (13.9)		24 (12.7)
Occasional	1587 (43.9)	1370 (44.9)	217 (38.2)		77 (40.7)
Frequent	1522 (42.1)	1250 (41.0)	272 (47.9)		88 (46.6)
Underlying disease, n (%)				<.001	
Present	701 (19.4)	637 (20.9)	64 (11.3)		20 (10.6)
Absent	2916 (80.6)	2412 (79.1)	504 (88.7)		169 (89.4)
Vaccination history, n (%)				<.001	
COVID-19 vaccine	2243 (62.0)	1967 (64.5)	276 (48.6)		93 (49.2)
Influenza vaccine	719 (19.9)	528 (17.3)	191 (33.6)		65 (34.4)
Other vaccine	295 (8.2)	226 (7.4)	69 (12.1)		24 (12.7)
Unvaccinated	360 (10.0)	328 (10.8)	32 (5.6)		7 (3.7)
Household fuel type, n (%)				.20	
Clean fuel	3295 (91.1)	2786 (91.4)	509 (89.6)		169 (89.4)
Traditional fuel	322 (8.9)	263 (8.6)	59 (10.4)		20 (10.6)
Cooking fume exposure, n (%)				.06	
Yes	3035 (83.9)	2573 (84.4)	462 (81.3)		152 (80.4)
No	582 (16.1)	476 (15.6)	106 (18.7)		37 (19.6)

a *P* values compare non–acute respiratory infection (ARI) and ARI groups.

b ILI: influenza-like illness.

### Time-Varying Exposures

The overall median annual mean PM_2.5_ concentration was 19.3 (IQR 14.3‐31.4) μg/m^3^, while the annual mean O_3_ concentration was 99.5 (IQR 98.4‐100.5) μg/m^3^(Table 2). Compared with participants without ARIs, those who developed ARIs were exposed to higher levels of PM_2.5_ but lower levels of O_3_. This pattern is likely attributable to seasonal variation, as ARI events predominantly occurred during the cold season when O_3_ concentrations are typically lower (Figure S2 in Multimedia Appendix 1). A similar pattern was observed for ILI, which also showed higher PM_2.5_ exposure and lower O_3_ levels.

**Table 2.** Distribution of time-varying environmental exposures and contextual factors by acute respiratory infections (ARIs) during follow-up.

Variables	Overall (N=3617)	Non-ARI (n=3049)	ARI (n=568)	*P* value^a^	ILI^b^ (n=189)
PM_2.5^c^_ (μg/m^3^), median (IQR)	19.3 (14.3‐31.4)	18.7 (14.2‐28.7)	24.3 (16.2‐36.9)	<.001	27.4 (16.4‐39.8)
O_3^d^_ (μg/m^3^), median (IQR)	99.5 (98.4‐100.5)	99.9 (98.8‐100.6)	96.2 (94.8‐97.3)	<.001	96.3 (94.9‐97.3)
Temperature (℃), median (IQR)	17.6 (17.6‐17.7)	17.6 (17.6‐17.7)	17.5 (16.9‐17.7)	<.001	17.5 (16.9‐17.7)
Relative humidity (%), median (IQR)	73.3 (72.6‐74.5)	73.1 (72.5‐74.0)	76.8 (75.7‐77.4)	<.001	76.9 (76.0‐77.4)
City-level ILI activity (%), median (IQR)	4.2 (4.2‐4.2)	4.2 (4.2‐4.2)	4.3 (3.7‐5.1)	<.001	4.7 (3.9‐5.3)
Season, n (%)				<.001	
Cold season	1470 (40.6)	1178 (38.6)	292 (51.4)		93 (49.2)
Warm season	2147 (59.4)	1871 (61.4)	276 (48.6)		96 (50.8)

a *P* values compare non–acute respiratory infections and ARIs groups.

b ILI: influenza-like illness.

c PM_2.5_: particulate matter 2.5.

d O_3_: ozone.

### Exposure Window Selection

The associations between air pollution exposure and ARIs or ILI across different exposure windows are presented in Table 3. For PM_2.5_, hazard ratios (HRs) tended to increase with longer exposure windows. For ARIs, the HR increased from 1.018 (95% CI 1.011-1.033) for the 3-month window to 1.031 (95% CI 1.023-1.039) for the 12-month window. For ILI, the HR increased from 1.021 (95% CI 1.011-1.031) for the 3-month window to 1.042 (95% CI 1.030-1.054) for the 12-month window. A similar pattern was observed for O_3_, with generally stronger associations observed under longer exposure windows and for ILI. On the basis of the lowest Akaike information criterion, the 12-month exposure window provided the best model fit for both pollutants and was selected for subsequent analyses.

**Table 3.** Associations of air pollutants with acute respiratory infections (ARIs) and influenza-like illness (ILI) across different exposure windows and comparison of model fit using the Akaike information criterion [AIC]).

Pollutant and exposure window (months)	ARI		ILI	
	HR^a^ (95% CI)^b^	AIC	HR (95% CI)^b^	AIC
PM_2.5_^c^				
3	1.018 (1.011‐1.033)	14,547.9	1.021 (1.011‐1.031)	6365.3
6	1.026 (1.018‐1.033)	14,505.9	1.031 (1.019‐1.042)	6312.0
9	1.029 (1.021‐1.037)	14,483.3	1.038 (1.026‐1.050)	6254.1
12	1.031 (1.023‐1.039)	14,479.0^d^	1.042 (1.030‐1.054)	6240.3^d^
O_3_^e^				
3	1.044 (1.008‐1.083)	14,666.3	1.087 (1.022‐1.157)	6499.8
6	1.123 (1.065‐1.183)	14,641.4	1.231 (1.127‐1.343)	6430.2
9	1.191 (1.115‐1.274)	14,617.3	1.372 (1.231‐1.530)	6346.5
12	1.262 (1.168‐1.363)	14,576.7^d^	1.446 (1.279‐1.636)	6313.7^d^

a HR: hazard ratio.

b Models were adjusted for age, sex, residence, household size, BMI, smoking status, alcohol consumption, mask-wearing frequency, underlying disease, vaccination history, household fuel, cooking fume exposure, season, and city-level influenza-like illness activity; hazard ratios represent per 1 μg/m3 increase.

c PM_2.5_: particulate matter 2.5.

d Indicates the lowest Akaike information criterion (best model fit) within each pollutant.

e O_3_: ozone.

### Main Associations

On the basis of the 12-month exposure window, both PM_2.5_ and O_3_ were associated with increased risks of ARIs, and consistent associations were observed for ILI across all exposure models (Table 4). For PM_2.5_, in the single-pollutant model, each IQR increase was associated with a significantly higher risk of ARIs (HR 1.691, 95% CI 1.428‐2.002) and ILI (HR 2.188, 95% CI 1.678‐2.852). After full adjustment for copollutants, temperature, and relative humidity, the associations remained statistically significant, with HRs of 1.594 (95% CI 1.340‐1.897) for ARIs and 1.948 (95% CI 1.484‐2.557) for ILI. For O_3_, in the single-pollutant model, each IQR increase was associated with HRs of 1.826 (95% CI 1.387‐2.405) for ARIs and 2.942 (95% CI 1.866‐4.640) for ILI. After full adjustment, the associations were slightly attenuated but remained statistically significant, with HRs of 1.510 (95% CI 1.135‐2.007) for ARIs and 2.229 (95% CI 1.385‐3.588) for ILI. Results per 1 μg/m^3^ increase were consistent in direction and statistical significance (Table S7 in Multimedia Appendix 1).

**Table 4.** Associations of air pollutants with acute respiratory infections (ARIs) and influenza-like illness (ILI) under different exposure adjustment models.

Pollutant and model	ARIs		ILI	
	HR^a^^,^^b^ (95%CI)^c^	*P* value	HR (95%CI)^c^	*P* value
PM_2.5_^d^				
Single pollutant	1.691 (1.428‐2.002)	<.001	2.188 (1.678‐2.852)	<.001
+O_3_^e^	1.587 (1.334‐1.887)	<.001	1.942 (1.481‐2.549)	<.001
+Temperature	1.592 (1.339‐1.894)	<.001	1.943 (1.481‐2.550)	<.001
+Humidity	1.594 (1.340‐1.897)	<.001	1.948 (1.484‐2.557)	<.001
O_3_				
Single pollutant	1.826 (1.387‐2.405)	<.001	2.942 (1.866‐4.640)	<.001
+PM_2.5_	1.521 (1.144‐2.021)	.004	2.219 (1.379‐3.571)	.001
+Temperature	1.509 (1.135‐2.006)	.005	2.226 (1.383‐3.583)	.001
+Humidity	1.510 (1.135‐2.007)	.005	2.229 (1.385‐3.588)	.001

a HR: hazard ratio.

b HRs represent per IQR increase (PM_2.5_=19.4 μg/m3; O_3_=2.5 μg/m3).

c Models were adjusted for age, sex, residence, household size, BMI, smoking status, alcohol consumption, mask-wearing frequency, underlying disease, vaccination history, household fuel, cooking fume exposure, season, and city-level ILI activity, with sequential additional adjustment for copollutant, temperature, and relative humidity.

d PM_2.5_: particulate matter 2.5.

e O_3_: ozone.

### Dose-Response and Subgroup Analyses

**Figure 1.**
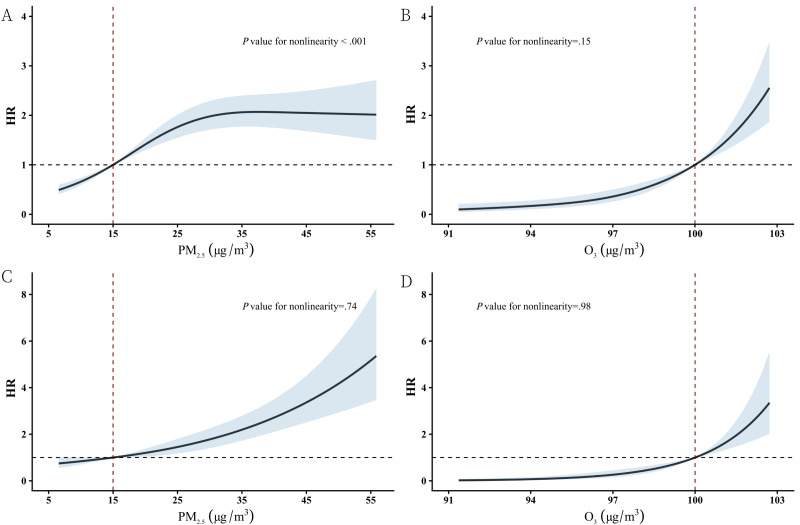
Dose-response relationships of long-term air pollutant exposure with acute respiratory infections (ARIs) and influenza-like illness (ILI): (A) and (B): ARIs and (C) and (D): ILI. Models were adjusted for age, sex, residence, household size, BMI, smoking status, alcohol consumption, mask-wearing frequency, underlying disease, vaccination history, household fuel, cooking fume exposure, season, and city-level ILI activity. Solid lines represent hazard ratios (HRs), and shaded areas indicate 95% CIs. The dashed horizontal line indicates HR=1. The red vertical line represents the World Health Organization (2021) guideline. O_3_: ozone; PM_2.5_: particulate matter 2.5.

On the basis of the WHO air quality guidelines, PM_2.5_ exhibited a nonlinear association with ARIs, characterized by a steeper increase in risk at lower concentrations followed by a plateau at higher concentrations. In contrast, linear exposure-response relationships were observed for PM_2.5_ with ILI and for O_3_ with both ARIs and ILI (Figure 1).

**Figure 2.**
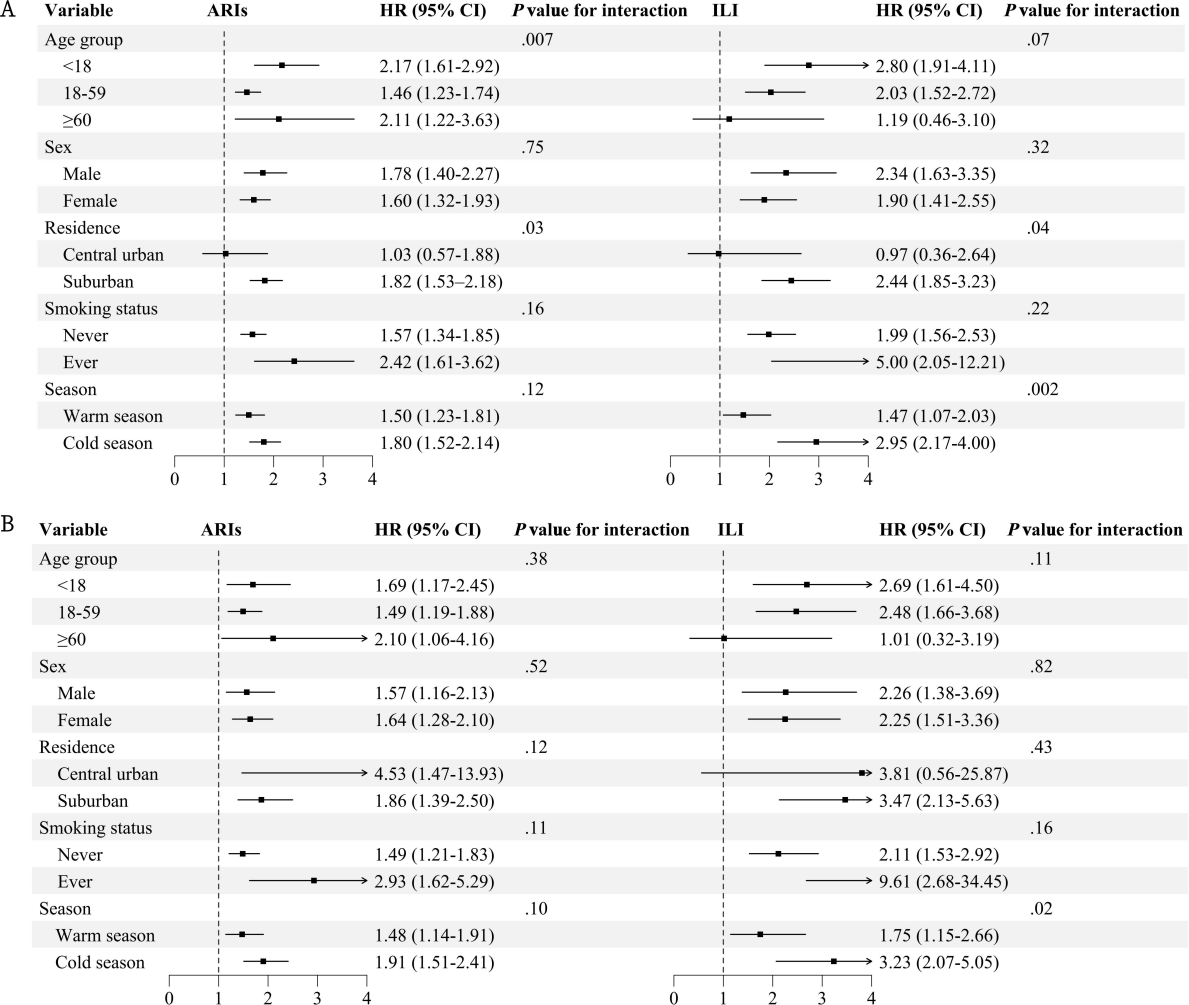
Subgroup analyses of the associations between long-term air pollution exposure and acute respiratory infections (ARIs) and influenza-like illness (ILI): (A) particulate matter 2.5 (PM_2.5_) and (B) ozone (O_3_). Models were adjusted for age, sex, residence, household size, BMI category, smoking status, alcohol consumption, mask-wearing frequency, underlying disease, vaccination history, household fuel, cooking fume exposure, season, and city-level ILI activity. Hazard ratios (HRs) represent per IQR increase (PM_2.5_=19.4 μg/m^3^; O_3_=2.5 μg/m^3^).

Subgroup analyses showed significant effect modification by age, residence, and season in the associations between PM_2.5_ and ARIs, with stronger associations observed among children, older adults, and suburban residents and during the cold season. No significant interactions were observed for sex or smoking status. For O_3_, a significant interaction with season was identified for ILI, with stronger associations in the cold season (Figure 2).

### Sensitivity Analyses

The associations of PM_2.5_ and O_3_ with ARIs and ILI were consistent across different modeling assumptions. Results remained robust when alternative recovery time definitions (7-day and 21-day definitions) and Andersen-Gill models were applied. Using ground-monitoring–based exposures yielded slightly stronger associations. In the subcohort, time-activity–weighted exposure strengthened PM_2.5_ associations, while O_3_ showed largely null effects (Tables S8-S12 in Multimedia Appendix 1).

## Discussion

### Principal Findings

On the basis of individual-level exposure assessment and high-frequency follow-up data, we found that long-term exposure to PM_2.5_ and O_3_ was associated with increased risks of ARIs, and similar associations were observed for ILI. These associations remained robust across different exposure windows, model adjustments, and multiple sensitivity analyses. PM_2.5_ showed a nonlinear association with ARIs, while linear relationships were observed for PM_2.5_ with ILI and O_3_ with both outcomes. In addition, effect modification was observed by age, residence, and season for PM_2.5_, while stronger effects of O_3_ were observed in the cold season.

### Comparison With Previous Studies

The incidence density of ARIs in this community-based population was approximately 0.27 per person-year, which was lower than that reported in similar community surveillance studies [25,26]. A 5-year community study in New York reported an incidence density of 0.62 per person-year, which may be partly explained by its broader case definition based on a single symptom [25]. In Hong Kong, the incidence density among older adults was approximately 0.9 per person-year, likely due to the older study population and a higher proportion of winter observations [26]. In some low-income countries, higher incidence rates have been reported, such as 2.66 per person-year in Uganda and up to 3.62 per person-year among older adults in India [27,28]. Differences in incidence density across studies may reflect variations in case definitions, participant age, follow-up duration, and health care accessibility.

We found that long-term exposure to PM_2.5_ and O_3_ was associated with increased risks of ARIs and ILI, consistent with previous epidemiological evidence. A nationwide cohort study in Denmark showed that PM_2.5_ exposure over different averaging periods (with incidence rate ratios ranging from 1.52 for a 1-month averaging period to 1.25 for a 12-month averaging period) was significantly associated with increased ARI risk [13]. However, O_3_ was negatively associated with infection risk in that study, which may be explained by its inverse spatial correlation with combustion-related pollutants such as nitrogen oxides. Another Danish prospective cohort further confirmed that long-term PM_2.5_ exposure over 3 years was associated with increased risks of both incident and recurrent LRIs, providing evidence across broader exposure windows [15]. A large Spanish cohort also demonstrated that long-term exposure to PM_2.5_ and O_3_ was associated with increased risks of LRIs, and these associations remained robust after accounting for residential mobility [11].

### Biological Mechanisms

Existing evidence suggests that the biological mechanisms underlying the effects of air pollution on respiratory infections mainly involve oxidative stress, inflammatory responses, and immune dysregulation. As fine PM capable of reaching the terminal bronchioles and alveolar regions, PM_2.5_ can deposit on the airway epithelium, induce reactive oxygen species production, and activate inflammatory signaling pathways such as nuclear factor kappa-light-chain-enhancer of activated B cells. This may promote the release of cytokines, including interleukin 6 and tumor necrosis factor [29]. Long-term exposure may also disrupt immune regulation, reduce the clearance of viruses and bacteria, and increase susceptibility to ARIs [30]. As a strong oxidant, O_3_ can deplete antioxidant defenses in the respiratory tract, induce chronic oxidative stress, damage ciliary structure, and impair mucociliary clearance. It may also contribute to airway remodeling and reduced lung function [31].

### Limitations

Several limitations should be acknowledged. First, exposure assessment was based on residential address–matched outdoor air pollution concentrations and did not account for indoor sources or individual time-activity patterns. Second, outcomes were based on symptom definitions without clinical confirmation, which may have introduced outcome misclassification and prevented differentiation between upper and lower respiratory tract infections. Finally, key confounders related to ARI risk, such as hand hygiene practices and exposure to health care settings, were not included in the covariate set, and residual confounding cannot be excluded.

### Conclusions

In conclusion, this prospective community-based study provides evidence that long-term exposure to PM_2.5_ and O_3_ is independently associated with increased risk of ARIs, with consistently stronger associations observed for ILI. Children and older adults emerged as particularly vulnerable subgroups, and the effects are more pronounced during cold seasons, underscoring the importance of targeted interventions for high-risk populations. These findings highlight the importance of long-term air pollution control and support targeted public health interventions for high-risk populations to reduce the burden of respiratory infections in the community. Individual mobility and indoor exposure should be considered in future exposure assessments, and the biological mechanisms linking long-term air pollution to ARIs should be further explored.

## Supplementary material

10.2196/88045 Multimedia Appendix 1 Supplementary tables and figures for sampling, variables, model diagnostics, and sensitivity analyses.

## Abbreviations

ARI: acute respiratory infection
HR: hazard ratio
ILI: influenza-like illness
LRI: lower respiratory infection
O_3_: ozone
PM: particulate matter
WHO: World Health Organization
