# Supplementary material for: Long-Term Exposure to Particulate Matter 2.5 and Ozone and the Risk of Acute Respiratory Infections: Community-Based Prospective Cohort Study
Source: JMIR Public Health Surveill. 2026 Jul 13;12:e88045. doi: 10.2196/88045 (PMC13362886; doi:10.2196/88045)
Supplement: Multimedia Appendix 1 [file publichealth-v12-e88045-s001.docx]

**Content**

[Table S1. Stratified random sampling framework by residence in Shanghai, China 2](#_Toc11759)

[Table S2. Definitions, classification and missing data handling of variables. 3](#_Toc12619)

[Table S3. Proportional hazards assumption tests for air pollutants in ARIs and ILI. (Schoenfeld residuals](#_Toc31451) *[P](#_Toc31451)*[-values). 4](#_Toc31451)

[Table S4. Crude hazard ratios of baseline characteristics for ARIs and ILI. 5](#_Toc2417)

[Table S5. Variance inflation factors for air pollutants and meteorological variables. 7](#_Toc10576)

[Table S6. Comparison of frailty and Andersen–Gill models for recurrent event analysis. 8](#_Toc32380)

[Table S7. Associations of air pollutants with ARIs and ILI under different exposure adjustment models (per 1 μg/m](#_Toc22925)^[3](#_Toc22925)^ [increase). 9](#_Toc22925)

[Table S8. Sensitivity analysis using a 7-day definition of recovery time. 10](#_Toc3536)

[Table S9. Sensitivity analysis using a 21-day definition of recovery time. 11](#_Toc24855)

[Table S10. Sensitivity analysis using Andersen–Gill models for recurrent events. 12](#_Toc5570)

[Table S11. Sensitivity analysis using ground monitoring station-based exposure assessment. 13](#_Toc12334)

[Table S12. Sensitivity analysis using time-activity weighted exposure assessment in sub-cohort. 14](#_Toc11213)

[Figure S1. Spatial distribution of residential locations and air pollution and weather stations in Shanghai, China. 15](#_Toc6470)

[Figure S2. Time-series of daily mean air pollutant exposure in participants. 16](#_Toc22424)

[References 17](#_Toc23277)

**Table S1. Stratified random sampling framework by residence in Shanghai, China**

| **Residence** | **Central urban** | |  | **Suburban** | | |
| --- | --- | --- | --- | --- | --- | --- |
|  | Jing'an | Xuhui |  | Songjiang | Fengxian | Jiading |
| Subdistricts/Towns, n (%) | 14（20.9） | 13（19.4） |  | 16（23.9） | 12（17.9） | 12（17.9） |
| Population Size, n (%) | 975 707（13.9） | 1 113 078（16.0） |  | 1 909 713（27.4） | 1 140 872（16.4） | 1 834 258（26.3） |
| Households, n (%) | 206（14.2） | 236（16.2） |  | 384  （26.4） | 243（16.7） | 385（26.5） |
| Recruited Participants, n (%) | 485（13.4） | 598（16.5） |  | 984  （27.1） | 580（16.0） | 984（27.1） |

**Table S2. Definitions, classification and missing data handling of variables.**

| **Variable** | **Definition / Measurement** | **Classification** | **Missing Data Handing** |
| --- | --- | --- | --- |
| Age | Date of birth | Continuous (years); grouped: <18, 18–59, ≥60 | None |
| Sex | Male or female | Binary | None |
| Height / Weight | Measured in person | Used to calculate body mass index | None |
| Body mass index (BMI) | Weight (kg) / Height² (m²) | Adults (≥18 years) ^[1]^: <18.5 underweight, 18.5–23.9 normal, 24.0–27.9 overweight, ≥28 obese;  Children and adolescents (2–17.9 years) ^[2]^: Age- and sex-specific cutoffs based on IOTF reference；  Infants (<2 years) ^[3]^: BMI-Z scores, <−2 SD underweight, −2 to +1 SD normal, >+1 to +2 SD overweight, >+2 SD obese. | None |
| Smoking status | Current smoking status | Current, former, never | Multiple imputation by chained equations |
| Alcohol consumption | Current frequency of alcohol consumption | Frequent, occasional, never | Multiple imputation by chained equations |
| Mask-wearing frequency | Frequency during daily life | Frequent, occasional, never | Multiple imputation by chained equations |
| Underlying disease | Self-reported | Dichotomized: Present vs. absent | Multiple imputation by chained equations |
| Vaccination history | Health record in past 3 years | COVID-19 vaccine, influenza vaccine, other vaccine (e.g., pneumococcal vaccine, MMR, DTP), coded as “unvaccinated” if no record | None |

**Table S3. Proportional hazards assumption tests for air pollutants in ARIs and ILI. (Schoenfeld residuals *P*-values).**

| **Pollutant** | **Window** | **ARIs** | **ILI** |
| --- | --- | --- | --- |
| PM_2.5_ |  |  |  |
|  | 3 months | .192 | .103 |
|  | 6 months | .181 | .049 |
|  | 9 months | .106 | .009 |
|  | 12 months | .173 | .016 |
| O_3_ |  |  |  |
|  | 3 months | .248 | .079 |
|  | 6 months | .227 | .006 |
|  | 9 months | .057 | .006 |
|  | 12 months | .118 | .068 |

**Table S4. Crude hazard ratios of baseline characteristics for ARIs and ILI.**

| **Characteristic** | **ARIs** | |  | **ILI** | |
| --- | --- | --- | --- | --- | --- |
|  | ***HR* (95%CI)** | ***P* value** |  | ***HR* (95%CI)^b^** | ***P* value** |
| Age group(y) |  |  |  |  |  |
| <18 | Reference |  |  | Reference |  |
| 18-59 | 0.464 (0.365-0.590) | <.001 |  | 0.362 (0.257-0.511) | <.001 |
| ≥60 | 0.211 (0.152-0.291) | <.001 |  | 0.175 (0.102-0.298) | <.001 |
| Sex |  |  |  |  |  |
| Male | Reference |  |  | Reference |  |
| Female | 1.430 (1.187-1.724) | <.001 |  | 1.328 (0.971-1.816) | .07 |
| Residence |  |  |  |  |  |
| Central urban | Reference |  |  | Reference |  |
| Suburban | 1.388 (1.124-1.715) | .002 |  | 1.688 (1.130-2.523) | .011 |
| Household size |  |  |  |  |  |
| 1 | Reference |  |  | Reference |  |
| 2 | 0.818 (0.572-1.170) | .27 |  | 1.275 (0.678-2.398) | .45 |
| ≥3 | 0.981 (0.709-0.359) | .91 |  | 1.407 (0.800-2.476) | .23 |
| BMI category(kg/m^2^) |  |  |  |  |  |
| Underweight | 1.525 (1.102-2.112) | .011 |  | 1.633 (1.080-2.469) | 0.020 |
| Normal | Reference |  |  | Reference |  |
| Overweight | 1.075 (0.855-1.351) | .54 |  | 0.783 (0.543-1.131) | .19 |
| Obese | 1.334 (0.921-1.933) | .12 |  | 1.226 (0.715-2.100) | .45 |
| Smoking status |  |  |  |  |  |
| Never | Reference |  |  | Reference |  |
| Ever | 0.484 (0.372-0.630) | <.001 |  | 0.406 (0.242-0.682) | <.001 |
| Alcohol consumption |  |  |  |  |  |
| Never | Reference |  |  | Reference |  |
| Ever | 0.646 (0.510-0.818) | <.001 |  | 0.609 (0.416-0.895) | .012 |
| Mask-wearing frequency |  |  |  |  |  |
| Never | Reference |  |  | Reference |  |
| Occasional | 0.950 (0.708-1.274) | .73 |  | 0.995 (0.628-1.578) | .98 |
| Frequent | 1.116 (0.839-1.484) | .45 |  | 1.024 (0.654-1.603) | .91 |
| Underlying disease |  |  |  |  |  |
| Absent | Reference |  |  | Reference |  |
| Present | 0.504 (0.377-0.674) | <.001 |  | 0.365 (0.235-0.568) | <.001 |
| Vaccination history |  |  |  |  |  |
| COVID-19 vaccine | 2.712 (2.204-3.338) | <.001 |  | 2.469 (1.758-3.467) | <.001 |
| Influenza vaccine | 2.670 (1.993-3.577) | <.001 |  | 2.967 (1.881-4.678) | <.001 |
| Other vaccine | 0.638 (0.435-0.933) | .021 |  | 0.331 (0.161-0.681) | .003 |
| Unvaccinated | Reference |  |  | Reference |  |
| Household fuel type |  |  |  |  |  |
| Clean fuel | Reference |  |  | Reference |  |
| Traditional fuel | 1.277 (0.947-1.720) | .10 |  | 1.277 (0.829-1.965) | .267 |
| Cooking fume exposure |  |  |  |  |  |
| Yes | 0.811 (0.639-1.030) | .08 |  | 0.765 (0.519-1.129) | .17 |
| No | Reference |  |  | Reference |  |
| Season |  | <.001 |  |  | <.001 |
| Warm season | Reference |  |  | Reference |  |
| Cold season | 1.489 (0.572-3.879) | .41 |  | 0.285 (0.032-5.549) | .26 |
| Abbreviations: ARIs, acute respiratory infections; ILI, influenza-like illness; BMI, body mass index; COVID-19, coronavirus disease 2019. | | | | | |

**Table S5. Variance inflation factors for air pollutants and meteorological variables.**

| **Variable** | **PM_2.5_** | **O_3 8h-max_** | **Temperature** | **Humidity** |
| --- | --- | --- | --- | --- |
| PM_2.5_ | – | 1.06 | 1.06 | 1.09 |
| O_3_ | 2.28 | – | 1.07 | 1.93 |
| Temperature | 4.26 | 1.99 | – | 2.01 |
| Humidity | 2.46 | 2.03 | 1.13 | – |

Abbreviations: PM_2.5_, particulate matter ≤2.5μm; O3, ozone.

**Table S6. Comparison of frailty and Andersen–Gill models for recurrent event analysis.**

| **Comparison** | **Frailty model** | **Andersen–Gill model** |
| --- | --- | --- |
| Purpose | Primary analysis | Sensitivity analysis |
| Key Features | Accounts for unobserved heterogeneity; captures subject-level random effects | Treats recurrent events as independent increments; flexible time-varying structure |
| Model Specification | Cox model with frailty term: coxph(Surv(start,stop,event) ~ X + frailty(id)) | Counting process model: coxph(Surv(start,stop,event) ~ X + cluster(id)) |
| Handling of Recurrent Events | Correlation modeled via random effects (frailty) | Recurrent events treated as repeated events; correlation adjusted via robust SE |
| Analytical Focus | Subject-specific effect (individual-level heterogeneity) | Population-averaged effect (overall risk) |

**Table S7. Associations of air pollutants with ARIs and ILI under different exposure adjustment models (per 1 μg/m^3^ increase).**

| **Pollutant** | **Model^a^** | **ARIs** | |  | **ILI** | |
| --- | --- | --- | --- | --- | --- | --- |
|  |  | ***HR* (95%CI)** | ***P* value** |  | ***HR* (95%CI)** | ***P* value** |
| PM_2.5_ |  |  |  |  |  |  |
|  | Single pollutant | 1.027 (1.019-1.036) | <.001 |  | 1.041 (1.027-1.055) | <.001 |
|  | + O_3_ | 1.024 (1.015-1.033) | <.001 |  | 1.035 (1.020-1.049) | <.001 |
|  | + Temperature | 1.024 (1.015-1.033) | <.001 |  | 1.035 (1.020-1.049) | <.001 |
|  | + Humidity | 1.024 (1.015-1.034) | <.001 |  | 1.035 (1.021-1.050) | <.001 |
| O_3_ |  |  |  |  |  |  |
|  | Single pollutant | 1.279 (1.143-1.431) | <.001 |  | 1.554 (1.290-1.872) | <.001 |
|  | + PM_2.5_ | 1.187 (1.057-1.333) | .004 |  | 1.385 (1.140-1.652) | .001 |
|  | + Temperature | 1.183 (1.053-1.329) | .005 |  | 1.387 (1.142-1.684) | .001 |
|  | + Humidity | 1.183 (1.053-1.329) | .005 |  | 1.388 (1.142-1.686) | .001 |

^a^ Models were adjusted for age, sex, residence, household size, BMI, smoking status, alcohol consumption, mask-wearing frequency, underlying disease, vaccination history, household fuel, cooking fume exposure, season, and city-level ILI activity, with sequential additional adjustment for co-pollutant, temperature, and relative humidity.

**Table S8. Sensitivity analysis using a 7-day definition of recovery time.**

| **Pollutant** | **Model^a^** | **ARIs** | |  | **ILI** | |
| --- | --- | --- | --- | --- | --- | --- |
|  |  | ***HR* (95%CI)** | ***P* value** |  | ***HR* (95%CI)** | ***P* value** |
| PM_2.5_ |  |  |  |  |  |  |
|  | Single pollutant | 1.716 (1.452-2.027) | <.001 |  | 2.031 (1.598-2.571) | <.001 |
|  | + O_3_ | 1.476 (1.243-1.753) | <.001 |  | 1.680 (1.311-2.154) | <.001 |
|  | + Temperature | 1.444 (1.214-1.718) | <.001 |  | 1.671 (1.301-2.143) | <.001 |
|  | + Humidity | 1.371 (1.151-1.634) | <.001 |  | 1.650 (1.277-2.131) | <.001 |
| O_3_ |  |  |  |  |  |  |
|  | Single pollutant | 3.250 (2.462-4.290) | <.001 |  | 5.129 (3.323-7.918) | <.001 |
|  | + PM_2.5_ | 2.764 (2.077-3.679) | <.001 |  | 4.259 (2.708-6.700) | <.001 |
|  | + Temperature | 2.351 (1.702-3.247) | <.001 |  | 3.726 (2.221-6.249) | <.001 |
|  | + Humidity | 1.816 (1.279-2.580) | .001 |  | 3.566 (2.403-6.225) | <.001 |

^a^ Models were adjusted for age, sex, residence, household size, BMI, smoking status, alcohol consumption, mask-wearing frequency, underlying disease, vaccination history, household fuel, cooking fume exposure, season, and city-level ILI activity, with sequential additional adjustment for co-pollutant, temperature, and relative humidity.

**Table S9. Sensitivity analysis using a 21-day definition of recovery time.**

| **Pollutant** | **Model^a^** | **ARIs** | |  | **ILI** | |
| --- | --- | --- | --- | --- | --- | --- |
|  |  | ***HR* (95%CI)** | ***P* value** |  | ***HR* (95%CI)** | ***P* value** |
| PM_2.5_ |  |  |  |  |  |  |
|  | Single pollutant | 1.716 (1.452-2.027) | <.001 |  | 2.068 (1.619-2.641) | <.001 |
|  | + O_3_ | 1.476 (1.243-1.753) | <.001 |  | 1.707 (1.325-2.198) | <.001 |
|  | + Temperature | 1.447 (1.221-1.713) | <.001 |  | 1.697 (1.316-2.189) | <.001 |
|  | + Humidity | 1.373 (1.157-1.630) | <.001 |  | 1.677 (1.292-2.177) | <.001 |
| O_3_ |  |  |  |  |  |  |
|  | Single pollutant | 3.301 (2.510-4.432) | <.001 |  | 5.416 (3.470-8.453) | <.001 |
|  | + PM_2.5_ | 2.813 (2.123-3.730) | <.001 |  | 4.495 (2.825-6.645) | <.001 |
|  | + Temperature | 2.411 (1.753-3.317) | <.001 |  | 3.908 (2.298-6.645) | <.001 |
|  | + Humidity | 1.877 (1.326-2.656) | <.001 |  | 3.747 (2.117-6.631) | <.001 |

^a^ Models were adjusted for age, sex, residence, household size, BMI, smoking status, alcohol consumption, mask-wearing frequency, underlying disease, vaccination history, household fuel, cooking fume exposure, season, and city-level ILI activity, with sequential additional adjustment for co-pollutant, temperature, and relative humidity.

**Table S10. Sensitivity analysis using Andersen–Gill models for recurrent events.**

| **Pollutant** | **Model^a^** | **ARIs** | |  | **ILI** | |
| --- | --- | --- | --- | --- | --- | --- |
|  |  | ***HR* (95%CI)** | ***P* value** |  | ***HR* (95%CI)** | ***P* value** |
| PM_2.5_ |  |  |  |  |  |  |
|  | Single pollutant | 1.663 (1.455-1.901) | <.001 |  | 2.224 (1.765-2.801) | <.001 |
|  | + O_3_ | 1.537 (1.332-1.773) | <.001 |  | 1.974 (1.565-2.489) | <.001 |
|  | + Temperature | 1.540 (1.335-1.777) | <.001 |  | 1.977 (1.566-2.496) | <.001 |
|  | + Humidity | 1.539 (1.335-1.774) | <.001 |  | 1.982 (1.573-2.498) | <.001 |
| O_3_ |  |  |  |  |  |  |
|  | Single pollutant | 2.055 (1.601-2.639) | <.001 |  | 3.180 (2.271-4.454) | <.001 |
|  | + PM_2.5_ | 1.734 (1.304-2.306) | <.001 |  | 2.571 (1.764-3.747) | <.001 |
|  | + Temperature | 1.726 (1.300-2.294) | <.001 |  | 2.559 (1.753-3.733) | <.001 |
|  | + Humidity | 1.726 (1.298-2.295) | <.001 |  | 2.560 (1.756-3.734) | <.001 |

^a^ Models were adjusted for age, sex, residence, household size, BMI, smoking status, alcohol consumption, mask-wearing frequency, underlying disease, vaccination history, household fuel, cooking fume exposure, season, and city-level ILI activity, with sequential additional adjustment for co-pollutant, temperature, and relative humidity.

**Table S11. Sensitivity analysis using ground monitoring station-based exposure assessment.**

| **Pollutant** | **Model^a^** | **ARIs** | |  | **ILI** | |
| --- | --- | --- | --- | --- | --- | --- |
|  |  | ***HR* (95%CI)^b^** | ***P* value** |  | ***HR* (95%CI)^b^** | ***P* value** |
| PM_2.5_ |  |  |  |  |  |  |
|  | Single pollutant | 4.226 (2.389-7.474) | <.001 |  | 5.162 (2.062-12.92) | <.001 |
|  | + O_3_ | 3.342 (1.885-5.923) | <.001 |  | 3.164 (1.252-8.000) | .01 |
|  | + Temperature | 3.268 (1.841-5.802) | <.001 |  | 2.963 (1.168-7.519) | .02 |
|  | + Humidity | 3.551 (1.969-6.403) | <.001 |  | 3.221 (1.239-8.371) | .02 |
| O_3_ |  |  |  |  |  |  |
|  | Single pollutant | 2.210 (1.851-2.639) | <.001 |  | 2.883 (2.132-3.897) | <.001 |
|  | + PM_2.5_ | 2.001 (1.645-2.435) | <.001 |  | 2.415 (1.732-2.366) | <.001 |
|  | + Temperature | 1.995 (1.637-2.431) | <.001 |  | 2.377 (1.701-3.320) | <.001 |
|  | + Humidity | 2.187 (1.678-2.851) | <.001 |  | 2.376 (1.700-3.322) | <.001 |

^a^ Models were adjusted for age, sex, residence, household size, BMI, smoking status, alcohol consumption, mask-wearing frequency, underlying disease, vaccination history, household fuel, cooking fume exposure, season, and city-level ILI activity, with sequential additional adjustment for co-pollutant, temperature, and relative humidity.

^b^ HRs represent per IQR increase (PM_2.5_ = 3.4 μg/m^3^; O_3_ = 2.7 μg/m^3^).

**Table S12. Sensitivity analysis using time-activity weighted exposure assessment in sub-cohort.**

| **Pollutant** | **Model^a^** | **ARIs** | |  | **ILI** | |
| --- | --- | --- | --- | --- | --- | --- |
|  |  | ***HR* (95%CI)^b^** | ***P* value** |  | ***HR* (95%CI)^b^** | ***P* value** |
| PM_2.5_ |  |  |  |  |  |  |
|  | Single pollutant | 1.594 (1.228-2.069) | <.001 |  | 2.370 (1.583-3.548) | <.001 |
|  | + O_3_ | 1.741 (1.333-2.273) | <.001 |  | 2.489 (1.644-3.768) | <.001 |
|  | + Temperature | 1.746 (1.337-2.280) | <.001 |  | 2.500 (1.651-3.787) | <.001 |
|  | + Humidity | 1.757 (1.345-2.293) | <.001 |  | 2.513 (1.657-3.811) | <.001 |
| O_3_ |  |  |  |  |  |  |
|  | Single pollutant | 0.813 (0.646-1.025) | .08 |  | 0.985 (0.687-1.413) | .93 |
|  | + PM_2.5_ | 0.726 (0.572-0.921) | .008 |  | 0.832 (0.573-1.208) | .33 |
|  | + Temperature | 0.724 (0.570-0.921) | .008 |  | 0.826 (0.569-1.199) | .32 |
|  | + Humidity | 0.721 (0.568-0.915) | .007 |  | 0.824 (0.567-1.196) | .31 |

^a^ Models were adjusted for age, sex, residence, household size, BMI, smoking status, alcohol consumption, mask-wearing frequency, underlying disease, vaccination history, household fuel, cooking fume exposure, season, and city-level ILI activity, with sequential additional adjustment for co-pollutant, temperature, and relative humidity.

^b^ HRs represent per IQR increase (PM_2.5_ = 14.5 μg/m^3^; O_3_ = 24.7 μg/m^3^).


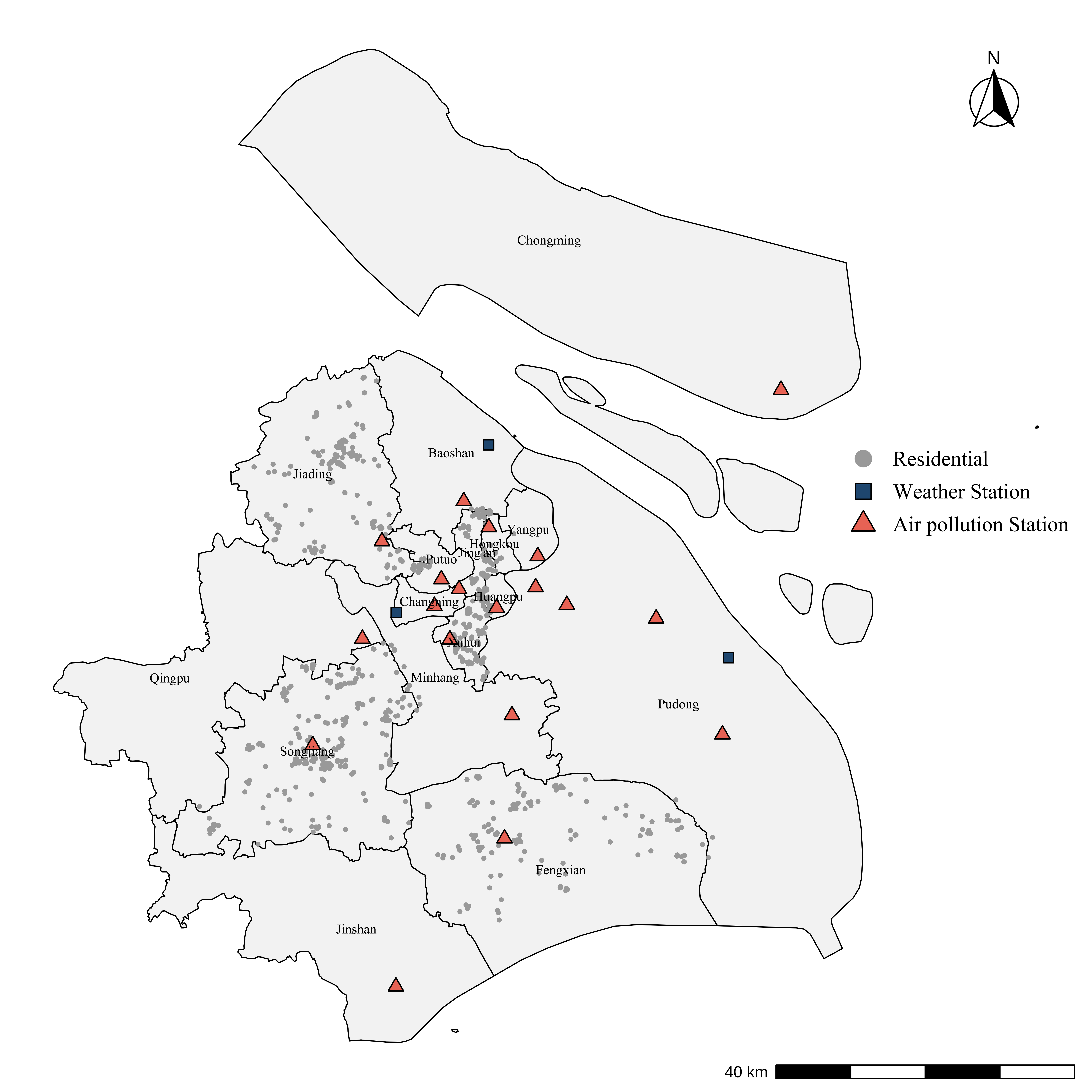


**Figure S1. Spatial distribution of residential locations and air pollution and weather stations in Shanghai, China.**

**
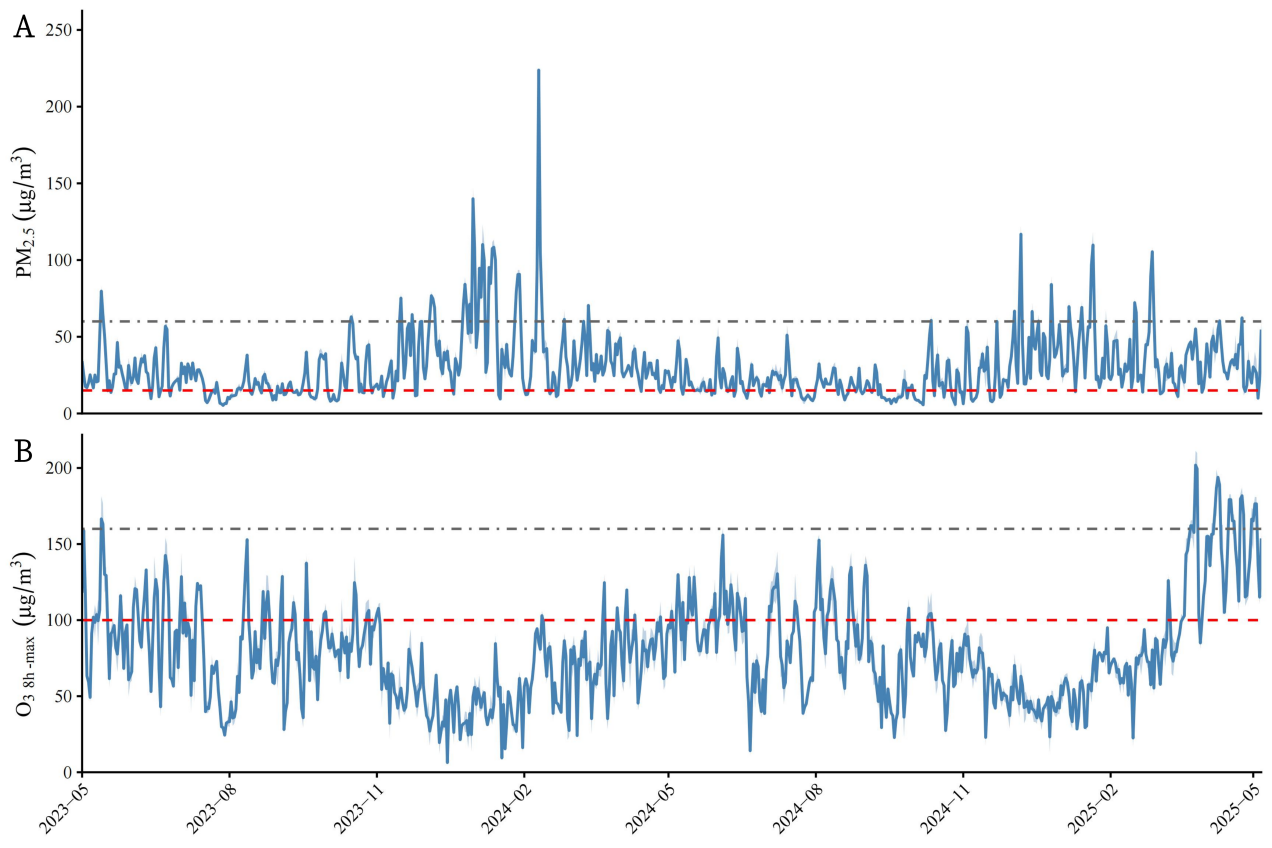
Figure S2. Time-series of daily mean air pollutant exposure in participants.**

Black lines: Chinese standards (GB3095-2012, Grade II): PM_2.5_ (75 μg/m^3^) and O_3_ 8-hour maximum (160 μg/m^3^); red lines: WHO (2021) guidelines: PM_2.5_ (15 μg/m^3^) and O_3_ 8-hour maximum (100 μg/m^3^).

**References**

1. Zhou BF; Cooperative Meta-Analysis Group of the Working Group on Obesity in China. Predictive values of body mass index and waist circumference for risk factors of certain related diseases in Chinese adults--study on optimal cut-off points of body mass index and waist circumference in Chinese adults. Biomed Environ Sci. 2002;15(1):83-96.
2. Cole TJ, Bellizzi MC, Flegal KM, et al. Establishing a standard definition for child overweight and obesity worldwide: international survey. BMJ. 2000;320(7244):1240-1243. doi:10.1136/bmj.320.7244.1240
3. World Health Organization. WHO child growth standards: height-for-age, weight-for-age, weight-for-length, weight-for-height and body mass index-for-age: methods and development. World Health Organization: Geneva, Switzerland, 2006.
